# Methods matter for dietary supplement exposure assessment: comparing prevalence, product types, and amounts of nutrients from dietary supplements in the Interactive Diet and Activity Tracking in the American Association of Retired Persons cohort study

**DOI:** 10.1016/j.ajcnut.2025.03.020

**Published:** 2025-04-02

**Authors:** Alexandra E Cowan-Pyle, Regan L Bailey, Jaime J Gahche, Johanna T Dwyer, Lindsay M Reynolds, Raymond J Carroll, Bani K Mallick, Diane C Mitchell, Janet A Tooze

**Affiliations:** 1 Department of Nutrition, Texas A&M University, College Station, TX, United States; 2 Department of Behavioral Sciences and Social Medicine, College of Medicine, Florida State University, Tallahassee, FL, United States; 3 NIH Office of Dietary Supplements, Bethesda, MD, United States; 4 Jean Mayer USDA Human Nutrition Research Center on Aging, Tufts University, Boston, MA, United States; 5 Department of Epidemiology and Prevention, Wake Forest School of Medicine, Wake Forest University, Winston-Salem, NC, United States; 6 Department of Statistics, Texas A&M University, College Station, TX, United States; 7 Institute for Advancing Health Through Agriculture, Texas A&M AgriLife Research, College Station, TX, United States; 8 Department of Biostatistics and Data Science, Wake Forest School of Medicine, Wake Forest University, Winston-Salem, NC, United States

**Keywords:** dietary supplement, dietary supplement assessment methods, IDATA, micronutrients, nutrient intake

## Abstract

**Background:**

Valid dietary supplement (DS) assessment methods are critical for nutrition research and monitoring as DS contributes substantially toward micronutrient exposures for millions of Americans. Little is known about how DS assessment tools vary in estimating the prevalence of use and micronutrient amounts from DS.

**Objectives:**

We compared repeat collections over a year of 2 commonly used DS assessment methods: the diet history questionnaire-II (DHQII) and the automated-self-administered 24-h dietary recall (ASA24), within the longitudinal Interactive Diet and Activity Tracking in American Association of Retired Persons (IDATA) study.

**Methods:**

DS information was collected among IDATA participants (*n* = 795; 50–74 y) who completed 2–6 ASA24s and a second DHQII. Agreement [Kappa (κ)] at the individual level and group-level prevalence of DS use (McNemar’s test) overall and by product type were compared among all participants. Mean calcium and vitamin D intakes, by source, and nutrient amounts per consumption day (i.e., dosages) from DS were compared between the DHQII and ASA24 among DS users. Calcium and vitamin D were chosen as priority nutrients, as they reflect vitamins and minerals and are ubiquitous in DS.

**Results:**

Prevalence of DS use varied by product type [13 of 28 comparisons differed in prevalence (McNemar’s test); Kappa agreement range: κ = –0.03 to 0.73)]. Mean consumption day amounts of vitamin D (but not calcium) were remarkably different as assessed by the DHQII and ASA24 (mean ± standard error): vitamin D ranged from 24 ± 2.7 to 45 ± 9.5 *μ*g/d on the ASA24 and from 12 ± 0.3 to 14 ± 0.3 *μ*g/d on the DHQII (*P* < 0.0001).

**Conclusions:**

Within IDATA, the comparability of ASA24 and DHQII in estimating the prevalence of use of and nutrient intakes from DS fluctuates by nutrient and product type. DS approaches beyond a questionnaire may be warranted for estimating absolute nutrient amounts, and the choice of the DS assessment method depends on the nutrient/dietary component of interest.

## Introduction

Accurately measuring all dietary exposures is critical to understanding how diet modulates health outcomes. To adequately understand these relationships, a complete assessment of foods, beverages, and dietary supplements (DSs) must be quantified, especially because DS use is highly prevalent in the United States [[1], [2], [3], [4], [5]]. Although measuring intakes of foods and beverages (F&B) is challenging [6] and well-documented, pervasive measurement error exists for energy reporting; measurement of DS should theoretically be more straightforward than F&B (i.e., often consumed in discrete doses) and unrelated to energy intake. However, the primary challenges of measuring F&B intakes include difficulties with memory, portion size estimation, and social desirability, all of which may differ for DS. Very limited research has been devoted to determining the best approaches for measuring DS use [7], intake amounts, and patterns of use, hampering our ability to understand the role of DS, if any, in human health.

Methods of DS assessment include food frequency questionnaires (FFQ), 24-h dietary recalls (24HR), product inventories, and records, which collectively have varying degrees of strengths and limitations [8,9]. Most longitudinal research studies use an FFQ to monitor habitual DS intakes, but existing FFQs ask a limited number of questions, are inconsistent in terms of the types of questions asked and the number of products that are queried on [10], and do not capture the complexities of DS product use over time. Although richer details on DS use can be garnered from recalls and records, the cost and time associated with their collection often preclude their use in large studies. The traditional 24HR is a better approximation of usual energy intakes, yet it is very costly and time-intensive when including DS reporting. The automated-self-administered 24HR (ASA24) was developed to provide a free resource that addresses the cost challenge of a 24HR [11], but DS assessment via ASA24 still adds a time burden for research participants.

Although DS is considered in scientific literature as either benign or having little impact, such conclusions cannot truly be drawn given the highly inconsistent methods for assessing DS use in longitudinal research, the short duration of clinical trials, and the various ways in which DS product types are defined. These limitations hinder optimal practices for DS assessment and have implications for research, monitoring, and policy settings. For this reason, we examined multiple DS assessment methods over the course of 1-y using the longitudinal Interactive Diet and Activity Tracking in the American Association of Retired Persons (IDATA) study, one of the most comprehensive studies to date with 2 commonly used DS methods collected at several time points [12]. This study aimed to compare an FFQ [i.e., a diet history questionnaire-II (DHQII)] and the mean of multiple 24HRs (i.e., ASA24), collected over a year, among adults (50–74 y; *n* = 795) in IDATA. This analysis focused on the prevalence of DS product reporting and intakes of calcium and vitamin D, as these nutrients are commonly consumed in the form of DS among adults in the United States [[13], [14], [15]], typically exhibit different DS usage patterns [14,15], are of public health relevance [16], and have been the topic of extensive discussion (i.e., in the context of DS) as of late [17].

## Methods

### IDATA background

The complete study details of IDATA have been published elsewhere but are briefly described below. Those adults (of both sexes) who preregistered on the study website or via telephone (*n* = 4967) participated in a prescreening telephone interview to determine if eligibility criteria were met (*n* = 3515). Eligible study participants (via prescreening) visited the study center for an in-person clinic visit, where demographic and biological data (i.e., sex, age, racial/ethnicity, height, weight, etc.) for each participant were collected (comprehensive detail on eligibility criteria is provided in Appendix 1), resulting in a final IDATA study sample of *n* = 1110 males and females aged 50–74 y. For this analysis, the analytic sample was further restricted to those with complete information on ≥2 ASA24s and the second DHQII (inclusive of complete DS information; *n* = 795); missing values were handled through exclusion from the analytic dataset to provide a complete dataset for the comparability analysis. A participant flowchart outlining sample identification in the current analysis using data from the IDATA cohort study is available in Supplemental Figure 1. For analyses specific to DS users, the analytic sample included only those who reported any DS use on the ASA24 method and/or the second DHQII (*n* = 709), as defined in Table 1; group-level differences between DS users and non-DS users are outlined in Table 2. Study approval was obtained via the National Cancer Institute (NCI) special studies institutional review board, and informed consent was obtained from all participants. Deidentified dietary and DS data were shared by the NIH/NCI for secondary data analysis under a data sharing agreement (cancer data access system #: CDAS-83), consistent with informed consent.

**TABLE 1:** Description of methods used for the calculation of the prevalence of dietary supplement use on automated self-administered 24-h dietary recall and the diet history questionnaire-II among the interactive diet and activity tracking in the American Association of Retired Persons study sample.

Identification of DS users	DHQII method	ASA24 method
Any DS	If the participant reported taking a “1-a-day, “Theragran-, Centrum-,” type product in the previous 12 mo, or taking “any vitamins, minerals, or other herbal supplements other than a MV” in the previous 12 mo on the DHQII.	If the participant reported taking any DS product in the previous 24 h on ASA24.
Calculation of prevalence of DS use for DS product types assessed		
MVM	If the participant reported taking a “1-a-day, “Theragran-, Centrum-,” type product in the previous 12 mo, and responded “yes” or “don’t know” to the MV containing minerals on the DHQII.	If the participant reported taking any DS product that contains ≥3 vitamins and ≥1 mineral.
MV	If the participant reported taking a “1-a-day, “Theragran-, Centrum-,” type product in the previous 12 mo and responded “no” to the MV containing minerals on the DHQII.	If the participant reported taking any DS product that contains ≥2 vitamins and does not contain any minerals.
Calculation of prevalence of DS use for DS product types assessed specifically for calcium		
Calcium from MVM	If the participant reported taking a “1-a-day, “Theragran-, Centrum-,” type product in the previous 12 mo, an MVM was assumed to include 162 mg calcium on the DHQII.	If the participant reported taking any DS product that contains ≥3 vitamins and ≥1 mineral and contains calcium. The amount of calcium in the MVM was assigned by the product on the ASA24.
Calcium and vitamin D DS	If the participant reported taking “calcium (with or without vitamin D) (not as part of a MV or antacid)” in the previous 12 mo, and “yes” to the calcium product containing vitamin D on the DHQII.	If the participant reported taking any DS product that contains both calcium and vitamin D as the primary ingredients and the product was not identified as an MVM.
Calcium from MM^1^	If the participant reported taking “calcium (with or without vitamin D) (not as part of a MV or antacid)” in the previous 12 mo and responded “yes” to the calcium product containing magnesium and/or zinc on the DHQII.	If the participant reported taking any DS product that contains calcium and ≥1 other mineral and does not contain any vitamins.
Calcium single nutrient DS	If the participant reported taking “calcium (with or without vitamin D) (not as part of a MV or antacid)” in the previous 12 mo and responded “no” to the calcium product containing vitamin D, magnesium, or zinc on the DHQII.	If the participant reported taking any DS product that contains only calcium and does not contain any other vitamins or minerals.
Calculation of prevalence of DS use for DS product types assessed specifically for vitamin D		
Vitamin D from MVM	If the participant reported taking a “1-a-day, “Theragran-, Centrum-,” type product in the previous 12 mo, an MVM was assumed to include 10 *μ*g of vitamin D on the DHQII.	If the participant reported taking any DS product that contains ≥3 vitamins and ≥1 mineral and contains vitamin D. The amount of vitamin D in the MVM was assigned by the product on the ASA24.
Calcium and vitamin D DS	If the participant reported taking “calcium (with or without vitamin D) (not as part of a MV or antacid)” in the previous 12 mo and responded “yes” to the calcium product containing vitamin D on the DHQII.	If the participant reported taking any DS product that contains both calcium and vitamin D as the primary ingredients and the product was not identified as an MVM.
Vitamin D from MV	If the participant reported taking a “1-a-day, “Theragran-, Centrum-,” type product in the previous 12 mo and responded “no” to the MV product containing minerals, an MV was assumed to include 10 μg of vitamin D on the DHQII.	If the participant reported taking any DS product that contains vitamin D and ≥1 other vitamin and does not contain any minerals.
Vitamin D single nutrient DS	If the participant marked “vitamin D” when asked to “please mark any of the following single supplements you took more than once per week” (not as part of a MV).	If the participant reported taking any DS product that contains only vitamin D and does not contain any other vitamins or minerals.

Abbreviations: ASA24, automated self-administered 24-h dietary recall; DHQII, diet history questionnaire-II; DS, dietary supplement; IDATA, interactive diet and activity tracking in American Association of Retired Persons; MM, multimineral; MV, multivitamin; MVM, multivitamin-mineral.

1 Multimineral DS was not directly assessed as a question on the DHQII in the IDATA study; therefore, for the purposes of the present analysis, the prevalence of use of multimineral DS products was assessed at a nutrient level (i.e., for calcium).

**TABLE 2:** Demographic and health-related characteristics of interactive diet and activity tracking in American Association of Retired Persons study sample^1^.

Characteristics	Total sample	DS users	Non-DS users
	Mean (SD) or *n* (%) *n* = 795	Mean (SD) or *n* (%) *n* = 709	Mean (SD) or *n* (%) *n* = 86
Sex			
Male, *n* (%)	406 (51.1)	347 (48.9)	59 (68.6)
Female, *n* (%)	389 (48.9)	362 (51.1)	27 (31.4)
Age (in years)	63.1 (5.9)	63.2 (5.9)	62.8 (5.8)
Age categories			
50–59 y	234 (29.4)	209 (29.5)	25 (29.1)
60–69 y	432 (54.3)	385 (54.3)	47 (54.6)
70+ y	129 (16.2)	115 (16.2)	14 (16.3)
Race and ethnicity, *n* (%)			
Non-Hispanic White	735 (92.5)	656 (92.5)	79 (91.9)
African American	52 (6.5)	49 (7.0)	3 (3.5)
Hispanic	3 (0.4)	1 (0.1)	2 (2.3)
Asian	5 (0.6)	3 (0.4)	2 (2.3)
BMI (kg/m^2^)	28.1 (4.6)	28.0 (4.6)	28.1 (4.2)

Abbreviations: ASA24, automated self-administered 24-h dietary recall; BMI, body mass index; DHQII, diet history questionnaire-II; DS, dietary supplement; IDATA, interactive diet and activity tracking in American Association of Retired Persons; SD, standard deviation.

1 Unless otherwise indicated, values are means ± SDs. The analytic sample includes IDATA participants aged 50–74 y who had complete information for the second DHQII and ASA24s. DS users were defined as those who reported any DS use on the ASA24 method and/or the second DHQII.

### IDATA study procedures

For data collection and study activities, IDATA participants were randomly assigned into 4 different study groups for dietary assignment sequencing and to reduce the dietary effects of seasonality. Each study group completed 3 study center visits using standardized procedures, and the timing of the data collection and activities completed varied between March 2012 and October 2013. Dietary data was predominantly collected using web-based platforms outside of the study center; however, participants were authorized to begin the web-based DHQII at the study center and complete it onsite or at home within 14 d of administration. For the ASA24, participants were asked to complete 6 bimonthly web-based administrations of the ASA24 (2011 version) on random days for 12 mo when prompted by email or telephone (i.e., self-administered during months 1, 3, 5, 7, 9, and 11). Each of these recalls was completed remotely and was self-administered. The second DHQII was used given that it queries dietary and DS intakes in the previous 12 mo and therefore covers the same time period in which the ASA24s were collected. A timeline schematic of all dietary assessment measurements in the IDATA study was previously published by Subar et al [12].

### Dietary assessment tools

The ASA24 is a fully automated, web-based dietary assessment tool developed by the NIH/NCI and is publicly available [11,18]. ASA24 can be used to capture 24HRs or food records for various research purposes and large-scale nutrition studies [11]. ASA24 guides the user through the 24HR using a multiple-pass approach based on the methodology of the USDA automated multiple-pass method to collect detailed information on intake of F&B. IDATA utilized the 2011 version of ASA24, in which participants were asked to recall and report all F&B consumed from midnight to midnight the previous day. A DS intake module is an included option of the ASA24 and was administered separately after completion of the 24HR dietary data collection. Dietary and DS databases (DSDs), including the USDA Food and Nutrient Database for Dietary Studies (version 4.1), MyPyramid Equivalents Database (version 2.0), and the NHANES-DSD 2007–2008, were integrated into ASA24 (2011 version) to estimate nutrients and other food components reported.

For the multiple collections of ASA24, respondents reported supplements they had consumed by either browsing through categories of supplements (e.g., multivitamin, botanical) or by searching for user-entered DS product names (e.g., calcium and vitamin D). Brand names and doses from the product label were included in the description for each product type. Participants were required to select the closest or exact match to the product they had consumed and report the quantity consumed for the day of intake. If the product type was unable to be located by the respondent, a text box for “unfound supplements” was available, where the participant could input the brand name or type of product, in addition to details on the amount consumed. This information was then provided to the researcher in the ASA24 output file, where it could be reviewed and matched with an NHANES-DSD supplement code based on the information provided, if possible [19].

In addition to ASA24*,* a FFQ developed by NIH/NCI, known as the web-based DHQII, was administered in the study center at month 1 and month 12; the present analysis utilized the second DHQII only as it queried the same time period as the ASA24. The DHQII was comprised of 134 food item questions and 8 questions on DS intake. Participants were also asked about the frequency of consumption (i.e., for F&B and DS) and portion size (i.e., for F&B) consumed over the previous 12 mo. For F&B, frequency of intake was reported using predefined categories ranging from “never” to “≥ 6 times per day” for beverages and from “never” to “≥2 times per day” for foods; portion size was selected based on 3 different portion size options.

For DS on the DHQII, information on whether a product was consumed was collected for 28 different product types (i.e., with yes/no responses only) for products consumed more than once per week; however, additional, more specific questions on the frequency of consumption (options range from: “<1 d/mo” to “every day”) and dose consumed (i.e., nutrient amount on the product label) were asked for only 5 product types, including multivitamins and calcium, iron, vitamin C, and vitamin E products. Additional information on product-specific questions and calculation of calcium and vitamin D amounts from DS on the DHQII is located in Appendix 1.

### Statistical analysis

Descriptive statistics (i.e., proportions and means) were used to describe the study sample overall and by DS use. Kappa statistics (κ), a measure of within-person agreement beyond that expected by chance [20], were utilized to evaluate *agreement* in the reported use of 28 DS product types between the ASA24 and the DHQII at the individual level. The κ was interpreted as follows: values ≤0 indicating no agreement, 0.01–0.20 as none to slight, 0.21–0.40 as fair, 0.41–0.60 as moderate, 0.61–0.80 as substantial, and 0.81–1.00 as almost perfect agreement [20]. McNemar’s test was used to determine if there were *differences in the prevalence of use [i.e., binary data (yes or no)]* of the 28 product types at the group level between DHQII and ASA24 methods; this statistic tests the null hypothesis that the prevalence of use of the product type of interest is equal between the 2 methods. Both the κ and McNemar test approaches were employed given that the κ considers the chance of agreement (i.e., sample size and statistical power) at the individual level, whereas the McNemar test evaluates differences in population-level prevalence estimates between methods.

Overall and specific product-level analyses for specific nutrients (i.e., calcium and vitamin D) were conducted for multivitamin-mineral (MVM), multivitamin (vitamin D only), multimineral (calcium only), calcium and vitamin D, and single nutrient DS product types. Classification of these DS product types at the micronutrient level is extensively discussed in Table 1. McNemar’s test was used to identify significant differences in the proportion of IDATA DS users who reported taking calcium and vitamin D from all DS product types and from MVMs by sex on the ASA24 and DHQII methods.

### Calcium and vitamin D

The estimated mean intake per consumption day (i.e., the amount the participant reported taking on a single day) from DS and the estimated mean nutrient intake (i.e., the mean amount of intake across all available days) from DS for calcium and vitamin D were calculated for DS users on each respective method. The estimated mean calcium and vitamin D intake from DS were then used to approximate total usual nutrient intakes from F&B and DS for each respective nutrient, as described in detail below. These estimates (i.e., mean intake per consumption day and mean nutrient intake from DS) were approximated to fully capture both episodic and habitual DS exposure to calcium and vitamin D because some DS product types (e.g., single vitamin D products) are only consumed occasionally in very high doses (e.g., 25,000 IU of vitamin D), whereas other product types are not. Extensive details on the calculation of mean nutrient intakes and mean intakes per consumption day from DS for calcium and vitamin D, as well as assigned default values for the ASA24 and DHQII methods, are in Appendix 1. Although specific sources of calcium and vitamin D in the diet (i.e., from F&B) were not examined in the present analysis, the primary dietary sources of these nutrients are well described elsewhere [21].

Mean total usual intakes of calcium and vitamin D from F&B and DS reported on the ASA24 among IDATA adult supplement users by the source of intake (i.e., F&B or DS product type) and sex were estimated using the “shrink then add” method; specifically, the NCI method was used to estimate usual intake from F&B (i.e., adjusted for random measurement error, such as within-person variation), and then the estimated mean intake from DS (i.e., for calcium and vitamin D, respectively) was added to the F&B intake to approximate total usual calcium and vitamin D intakes on the ASA24 [8,[22], [23], [24]]. For the DHQII, estimated mean nutrient intakes for calcium and vitamin D from F&B reported on the DHQII were added to the estimated mean calcium and vitamin D intake from DS to approximate the total usual intake for calcium and vitamin D, respectively.

**FIGURE 1:**
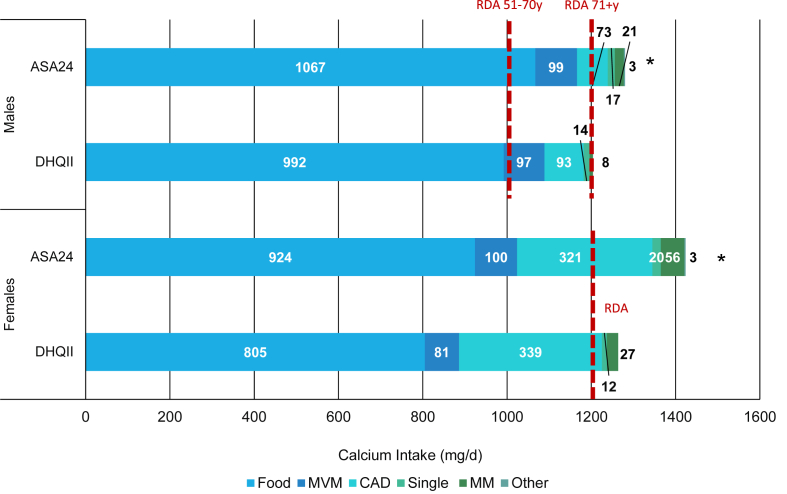
Mean total usual calcium intake reported on the DHQII or ASA24s among IDATA adult supplement users by calcium source and sex (*n* = 709)^1,2^. ASA24, automated self-administered 24-h dietary recall; CAD, calcium and vitamin D supplement; DHQII, Diet history questionnaire-II; DS, dietary supplement; IDATA, interactive diet and activity tracking in American Association of Retired Persons; MVM, multivitamin-mineral; MM, multimineral; Other, other DS sources; RDA, recommended dietary allowance; Single, single nutrient DS. ^1^ Values inside bars are means. The analytic sample includes individuals aged 50–74 y who had complete information for the second DHQII and ASA24s and reported DS intake on the DHQII or on an ASA24. The RDA values for calcium intake are 1000 mg/d among males 51–70 y, 1200 mg/d among males 71+ y, and 1200 mg/d among females 51–70 y and 71+ y. ^2^The asterisk indicates a significant difference in estimated mean calcium intake within sex, as compared to the ASA24 method (if applicable). A Bonferroni-corrected *P* value <0.0083 was considered statistically significant.

**FIGURE 2:**
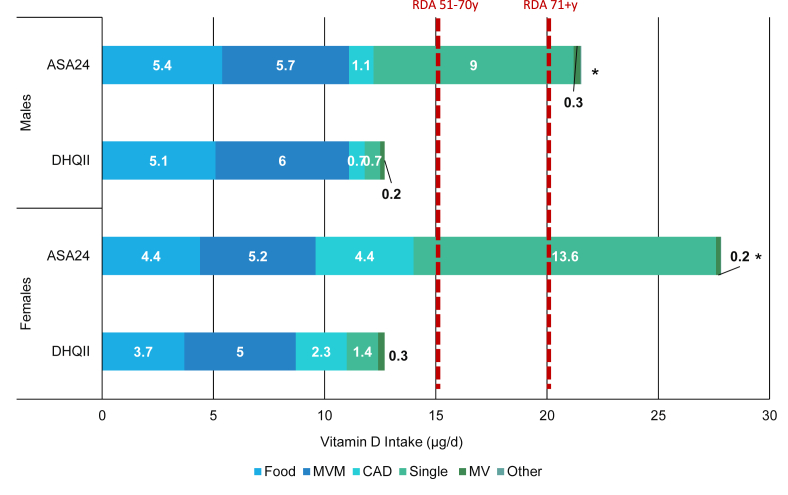
Mean total usual vitamin D intake reported on the DHQII or ASA24s among IDATA adult supplement users, by vitamin D source and sex (*n* = 709)^1,2^. ASA24, automated self-administered 24-h dietary recall; CAD, calcium and vitamin D supplement; DHQII, diet history questionnaire-II; DS, dietary supplement; IDATA, interactive diet and activity tracking in American Association of Retired Persons; MVM, multivitamin-mineral; MV, multivitamin; Other, other DS sources; RDA, recommended dietary allowance; Single, single nutrient DS. ^1^Values inside bars are means. The analytic sample includes individuals aged 50–74 y who had complete information for the second DHQII and ASA24s and reported DS intake on the DHQII or an ASA24. The RDA values for vitamin D intake are 15 *μ*g/d among males and females 51–70 y and 20 *μ*g/d among males and females 71+ y. ^2^The asterisk indicates a significant difference in estimated mean vitamin D intake, as compared to the DHQII method within sex. A Bonferroni-corrected *P* value <0.0083 was considered statistically significant.

**FIGURE 3:**
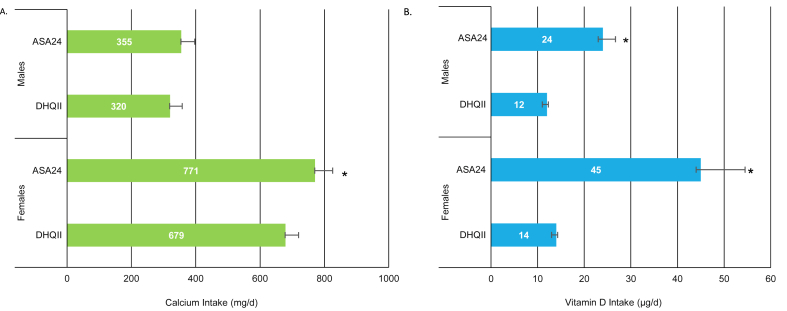
Mean calcium and vitamin D intake per consumption day from DS only reported on the DHQII or an ASA24 among IDATA adult supplement users, by sex (*n* = 709)^1–3^. ASA24, automated self-administered 24-h dietary recall; DHQII, diet history questionnaire-II; DS, dietary supplement; IDATA, Interactive Diet and Activity Tracking in American Association of Retired Persons. ^1^The panels reflect the mean DS intake per consumption day of calcium (A) and vitamin D (B) reported on the DHQII or an ASA24 among IDATA supplement users. ^2^Unless otherwise indicated values are means. The analytic sample includes individuals aged 50–74 y who had complete information for the second DHQII and ASA24s and reported DS intake on the DHQII or ASA24s. ^3^The asterisk indicates a significant difference in estimated calcium or vitamin D intake per consumption day from DS, as compared to that of the DHQII method. A Bonferroni-corrected *P* value <0.0083 was considered statistically significant.

Differences in estimated total usual nutrient intake from F&B + DS (as presented in FIGURE 1, FIGURE 2) and mean nutrient intake per consumption day from DS (as presented in Figure 3) reported on the DHQII and ASA24 methods (i.e., for calcium and vitamin D), respectively, were compared using 2-group t-tests. Statistical significance was set at a Bonferroni-corrected *P* value of 0.0083 to account for the multiplicity of comparisons (unless otherwise noted). All analyses were performed using SAS software (version 9.4; SAS Institute Inc).

## Results

Among the DS product types reported on the ASA24 and DHQII methods, prevalence of use at the group level significantly differed between methods for 13 out of 28 products assessed, including multivitamin (ASA24: 21% compared with DHQII: 3%), calcium multimineral (ASA24: 8% compared with DHQII: 14%), potassium (ASA24: 5% compared with DHQII: 3%), vitamin B complex (ASA24: 11% compared with DHQII: 8%), and vitamin C (ASA24: 17% compared with DHQII: 27%), vitamin D (ASA24: 74% compared with DHQII: 77%), and vitamin E (ASA24: 10% compared with DHQII: 15%; all *P* ≤ 0.006) micronutrient-containing products, as well as ω-3 (ASA24: 42% compared with DHQII: 32%), fish oil (ASA24: 42% compared with DHQII: 30%), flaxseed oil (ASA24: 1% compared with DHQII: 6%), joint (ASA24: 21% compared with DHQII: 13%), probiotic (ASA24: 5% compared with DHQII: 8%), and sport (ASA24: 5% compared with DHQII: 1%; all *P* < 0.001) nonmicronutrient containing DS (Supplemental Table 1; Table 3). Kappa agreements (κ), reflecting the agreement of DS exposure within individuals, ranged from 0.03 (multivitamin), showing almost no agreement, to 0.73 (vitamin D), showing a substantial agreement (Table 3). In contrast, most micronutrient-containing products (i.e., 13 out of 18 micronutrient-containing DS) exhibited a moderate or substantial agreement (all κ ≥ 0.50).

**TABLE 3:** Prevalence of use and agreement of micronutrient-containing product types on the automated self-administered 24-h dietary recall and diet history questionnaire-II methods among the interactive diet and activity tracking in American Association of Retired Persons study sample (*n* = 795)^1^.

	Total sample	Group-level prevalence on ASA24	Group-level prevalence on DHQII	Individual-level agreement	Comparison of group-level prevalence
	*n*	*n* (%)	*n* (%)	Kappa^2^	*P* value^3^
*Product Type*					
Multivitamin	795	169 (21.3)	25 (3.1)	0.03	<0.001
MVM	795	484 (60.9)	461 (58.0)	0.71	0.030
Calcium	795	564 (70.9)	552 (69.4)	0.66	0.257
Calcium and vitamin D	795	276 (34.7)	254 (31.9)	0.70	0.033
Calcium MM	795	60 (7.5)	107 (13.5)	0.38	<0.001
Iron	696	38 (5.5)	49 (7.0)	0.67	0.034
Vitamin A	795	9 (1.1)	12 (1.5)	0.18	0.467
Vitamin D	795	586 (73.7)	613 (77.1)	0.73	0.002
Vitamin C	696	121 (17.4)	188 (27.0)	0.63	<0.001
Vitamin E	696	72 (10.3)	106 (15.2)	0.63	<0.001
Vitamin B12	795	85 (10.7)	67 (8.4)	0.55	0.022
Vitamin B6	795	15 (1.9)	19 (2.4)	0.58	0.285
Vitamin B Complex	795	88 (11.1)	62 (7.8)	0.35	0.006
Folic acid/folate	795	22 (2.8)	20 (2.5)	0.51	0.655
Magnesium	795	60 (7.5)	58 (7.3)	0.67	0.739
Potassium	795	38 (4.8)	24 (3.0)	0.63	0.003
Selenium	795	5 (0.6)	6 (0.8)	0.18	0.739
Zinc	795	26 (3.3)	24 (3.0)	0.50	0.683

Abbreviations: AASA24, automated self-administered 24-h dietary recall; DHQII, diet history questionnaire-II; DS, dietary supplement; MM, multimineral; MVM, multivitamin-mineral.

1 Unless otherwise indicated, values are percentages (%). The analytic sample includes individuals aged 50–74 y who had complete information for the second DHQII and ASA24s.

2 Kappa (κ) reflects the agreement of DS product types on the ASA24 and the DHQII at the individual level. The κ was interpreted as follows: values ≤0 (no agreement); 0.01–0.20 (none to slight agreement), 0.21–0.40 (fair agreement), 0.41– 0.60 (moderate agreement), 0.61–0.80 (substantial agreement), and 0.81–1.00 (almost perfect agreement).

3 *P* value indicates a significant difference in estimated group-level prevalence (i.e., via McNemar’s test) between the DHQII and ASA24 methods. A Bonferroni-corrected *P* value <0.00625 was considered significant.

When estimating mean total usual calcium intake by source of intake among DS users, the source of the majority of intake originated from diet alone, with dietary intakes (i.e., from F&B; mean ± SE) ranging from 805 ± 21.4 mg/d (females) to 992 ± 28.0 mg/d (males) from the DHQII, and 924 ± 18.2 mg/d (females) to 1067 ± 21.0 mg/d (males) from the ASA24 (Figure 1). Total calcium intake (i.e., from F&B + DS) was higher using the ASA24 method for both males and females (*P* < 0.0083). For intakes from DS only, mean calcium intake by source (e.g., multimineral, calcium and vitamin D, MVM DS) significantly differed by method for males and females. Most notably, for females, the largest proportion of calcium from DS was derived from calcium and vitamin D supplements [mean ± SE: 321 ± 20.2 mg/d (ASA24) to 339 ± 20.6 mg/d (DHQII)], but for males, intakes of calcium from MVMs [97 ± 4.1 mg/d (ASA24) to 99 ± 4.8 mg/d (DHQII)] and calcium and vitamin D products [73 ± 11.2 mg/d (ASA24) to 93 ± 13.7 mg/d (DHQII)] were generally comparable. By method, among males, the estimated calcium intake from MVMs reported from the DHQII and ASA24 was similar (ASA24: 97 ± 4.1 mg/d compared with DHQII: 99 ± 4.8 mg/d), whereas among females, reported intake was higher from the ASA24 (ASA24: 100 ± 6.0 mg/d compared with DHQII: 81 ± 4.0 mg/d; *P* < 0.0001). Notably, mean intakes from diet alone only met the recommended dietary allowance for males ages 51–70 y (mean intake of males 51–70 y: 1067 ± 21.0 mg/d; recommended dietary allowance: 1000 mg/d) (Figure 1).

Unlike calcium, the majority of usual total vitamin D intake among DS users did not originate from the diet but rather came from several types of DS products (Figure 2). Intakes from DS differed markedly by method for both males and females, with mean vitamin D intakes from DS (mean ± SE) ranging from 7.7 ± 0.3 *μ*g/d (males) and 9.0 ± 0.3 *μ*g/d (females) on the DHQII, to 16.1 ± 2.2 *μ*g/d (males) and 23.4 ± 2.6 *μ*g/d (females) (*P* < 0.0001) on the ASA24, respectively. The largest proportion of intake from DS was contributed by single vitamin D DS; the estimated vitamin D intake from single vitamin D products varied considerably by method, with reportedly higher intakes on the ASA24 (9 ± 2.1 to 13.6 ± 2.6 *μ*g/d) than the DHQII for both males and females (0.7 ± 0.1 to 1.4 ± 0.1 *μ*g/d; both *P* ≤ 0.0001) (Figure 2). MVMs and calcium and vitamin D products were other major sources of vitamin D intake from DS; this was especially true among females. As was the case for single vitamin D products, the amounts of calcium from calcium and vitamin D DS were higher on the ASA24 method when compared with that of the DHQII for both sexes, yet the contributions from MVMs to overall vitamin D intake from DS were comparable across methods (Figure 2).

Among DS users, mean calcium intake per consumption day from DS (i.e., dosages on the days on which they consumed DS) did not vary between methods for males, but a differential pattern was observed for females (Figure 3); the estimated mean consumption day amount from DS (mean ± SE) among females was ∼771 ± 27.9 mg/d from the ASA24, but only 679 ± 20.8 mg/d from the DHQII (*P* < 0.0001). For vitamin D, estimated mean consumption day amounts from DS differed by DS reporting method regardless of sex, with intakes significantly higher on the ASA24 method (24 ± 2.7 to 45 ± 9.5 *μ*g/d) when compared with that of the DHQII method (12 ± 0.3 to 14 ± 0.3 *μ*g/d), for both males and females (*P* < 0.0001) (Figure 3).

Among DS users, the prevalence of use of any products that contain calcium or vitamin D, as well as within specific product types, such as MVM, were generally comparable from the DHQII and ASA24 for both males and females who report taking a DS (Supplemental Table 2). For example, among male DS users, 80% and 82% reported taking a vitamin D-containing DS product on the ASA24 and DHQII methods, respectively (Supplemental Table 2); whereas ∼85% of female DS users reported taking a vitamin D-containing DS on the ASA24, and 90% did so on the DHQII (Supplemental Table 2).

## Discussion

A call to action over 20 y ago [25] urged that a concerted effort was desperately needed to improve DS data collection and analysis, as DS data are critical to valid, accurate, and complete nutritional exposure classification [26]; this call remains highly relevant today. The 2 DS assessment tools compared in this study led to very different prevalence estimates for certain product types and different nutrient amounts, highlighting that the method used to quantify DS exposures greatly influences the population-level estimates obtained. This phenomenon has also been noted in comparisons of other DS assessment methods, and differences are amplified even more so when examined by sex [14,27,28]. Additionally, within-person agreement varied considerably by DS product type and was only moderate for some single nutrient products, indicating that the DS instrument is also likely to misclassify DS exposure at the individual level. Although other data suggest that DS exposures vary by method [27,28], the available literature is limited in the number of studies conducted [14,[27], [28], [29], [30], [31]], small sample sizes, and most are dated. Thus, it is surprising, given the pervasive use of DS [[1], [2], [3], [4],^5^,^32^], that the best method(s) for characterizing DS intakes remains unknown [[7], [8]]. Nevertheless, the limited collective knowledge [14,[27], [28], [29], [30], [31]], together with this study, indicates that the choice of the DS measurement tool used is impactful for assessing total nutrient exposures, which has implications for interpreting diet and health relationships and in nutrition surveillance and monitoring [7]. As the food landscape and the availability of supplement formulations in the marketplace continue to evolve, timely, innovative research with an emphasis on rigor and precision is greatly needed.

The “gold-standard” method for measuring DS use is the product inventory, in which trained interviewers record all DS information on use in the prior 30 d directly from the product container [33]; but, given the cost and time burden required, such a rigorous method is not widely used in large-scale research settings. Moreover, there are no recovery biomarkers or “truth” when examining DS exposures [34], so even the use of an inventory is unable to reflect true validity and/or reliability [35].

A recent study using the 2011–2014 NHANES [14] compared 4 approaches for characterizing DS use constructed from the 24HR and the in-home inventory among adults in the United States and determined that a higher percentage of adults reported DS use when the in-home inventory was combined with ≥1 24HR (57%), as compared to the use of either method alone (52% inventory; 43% 24HR). This considerable variation in DS use estimates may arise from the inventory’s ability to account for frequency-based information (i.e., over ∼30 d), detecting more intermittent DS use than 24HRs [14]. Without frequency-based information, supplemental intakes may be overestimated at the individual level, leading to exposure misclassification and an inaccurate representation of the full complexity of nutrient exposures [8,14]. Nonetheless, >500 DS users reported new products consumed between the in-home inventory and the 24HRs, indicating that interpretation of findings can be affected by the method used, and the use of both short- and long-term assessment tools may potentially capture a larger proportion of DS users, providing a more robust estimate than the use of either assessment tool alone [14]. However, much more work is needed to ensure that the most accurate and reliable methods of DS assessment are identified and accessible to investigators.

### Vitamin D as a case study

In this IDATA analysis, the differences in mean vitamin D exposure amounts (i.e., the sum of the frequency of use and consumption day amount) from DS were particularly striking (∼45% difference in the mean amounts on the ASA24 compared with DHQII). This may be, in part, due to physician-prescribed single vitamin D products that are used in high doses once per week, as opposed to other DS product types (e.g., MVMs) that are commonly sold over the counter and are more habitually consumed. Other studies comparing mean vitamin D intakes across DS assessment methods also show vast differences in the nutrient amounts obtained depending on the method used [14,27,28]. The most rigorous study conducted to date, the Multi-Ethnic Cohort Supplement Reporting Study (n = 1029; mean age of 68 y), suggested that questionnaire data can only be used to correctly rank nutrients at the group level (compared with the product inventory) and that vitamin D amounts obtained via questionnaire underestimated supplemental vitamin D by 20% overall, with higher amounts for females (32%) than males (7%) [27]. These findings align with the present study and highlight the importance of selecting DS instruments suited to the purpose of the study.

### Strengths and limitations

Although a strength of IDATA is that several repeat 24HRs (i.e., ≤6) were collected, the 24HRs were self-administered via ASA24 and completed online in the participant’s home. Similarly, the DHQII was administered online during the clinic visit with the opportunity to finish the questionnaire at home; thus, variability in data collection settings may have affected the resultant findings on the DHQII. Any study utilizing self-reported DS data has inherent caveats to consider, including the limited diversity of the study sample, limitations associated with self-report dietary data, and the measurement error structure of DS reporting for each instrument [8]. Calcium and vitamin D were prioritized for this analysis as they reflect both habitual and episodic DS use and are commonly found in supplemental sources; nonetheless, the comparability of intake estimates reported on 24HRs and FFQs may vary for other nutrients. The IDATA study sample is neither nationally representative nor generalizable to other racial and/or ethnic groups beyond non-Hispanic Whites. In IDATA, ∼89% of older adults (50–74 y) reported DS use; yet, in NHANES, only ∼70% of United States older adults (≥60 y) did so [32]; these differential patterns of DS use were anticipated, as DS use varies within adult population subgroups and IDATA participants reflect a high DS user population (i.e., older, non-Hispanic White, American Association of Retired Persons members).

Despite these limitations, research of this nature is critical to advancing our understanding of nutrient exposures and promoting the development of novel techniques with increased accuracy and precision. Currently, no consensus exists on the best method for measuring DS intake. The comparability of 24HRs and FFQs in measuring the prevalence of use of and estimating nutrient intakes from DS varies by the nutrient of interest, the source of intake, and the type of DS product consumed. Research and guidance involving DS exposures must be interpreted with the limitations of the instruments used in mind and utilize multiple assessment strategies capturing both habitual and episodic DS.

In conclusion, DS assessment can be completed using a variety of tools. In general, methods that have the capability to query specific product-level details perform better than a finite list of questions [8,27,35]. FFQs are commonly used in epidemiological studies, but they lack the requisite information necessary to estimate mean nutrient exposures, often rely on default amounts, and only query a limited number of products [35]; these limitations may drive the vast differences in estimates obtained between 24HRs and FFQs for some nutrients. Thus, understanding the role of databases, data quality, confounding, and biases in discrepant findings is critical in any intake analysis; however, considering the assessment tool used and its associated measurement error is equally as important. FFQs have good reproducibility for estimating the *prevalence* of MVM use in some cohorts and, in turn, may be suitable for obtaining basic DS information (e.g., yes/no responses). However, for studies that aim to estimate actual *amounts* of nutrients, it is vital that novel, robust DS methods (e.g., inventories) beyond an FFQ that have been tested across time in various cohorts are employed. As precision nutrition evolves, quantifying absolute nutrient amounts is necessary for tailored dietary guidance – particularly when episodically consumed nutrients (e.g., vitamin D) that demand precise DS tools for accurate estimates are of interest. This study, and the work of others [[27], [28], [29], [30], [31]], illustrate that it is crucial that the selection of the DS assessment instrument is chosen and interpreted with the known limitations of each method kept firmly in mind.

## Author contributions

The authors’ responsibilities were as follows – JAT: designed the research and concepts presented; AEC-P, RLB: consulted on the concepts presented and the analytical plan; AEC-P, RLB, JAT: wrote sections of the paper; AEC-P, JAT: completed the analysis; RLB, JJG, JTD, LMR, DCM, RJC, BKM, JAT: provided critical review and insights presented; and all authors: read and approved the final manuscript.

## Data availability

Deidentified data described in this manuscript is provided by the National Cancer Institute (NCI) under an NIH/NCI data sharing agreement for the interactive diet and activity tracking in the American Association of Retired Persons study [cancer data access system (CDAS) #: CDAS-83]. The code book and analytic code will be made available upon request, pending application and approval, under the conditions of an approved NIH/NCI CDAS data sharing agreement.

## Funding

This research was funded by an NIH/National Cancer Institute grant (NIH/NCI 2U01CA215834-05A1). Any opinions, findings, conclusions, or recommendations expressed in this publication are those of the author(s) and do not necessarily reflect the view of any entity within the NIH. The funders had no role in the design of this comparison study or in the interpretation of the data.

## Conflict of interest

AEC-P reports a relationship with the *Journal of the Academy of Nutrition and Dietetics*, including board membership, and with the National Pork Board, including consulting or advisory. RLB reports relationships involving consulting or advisory with the NIH Office of Dietary Supplements, Nestlé, General Mills Inc. Bell Institute, RTI International, Think Healthy Group, LLC, and Nutrition Impact, LLC. She also reports board memberships with the International Food Information Council, the International Life Sciences Institute (formerly), and the *Journal of Nutrition* *(formerly)*. RLB has received travel support in the past to present her research on dietary supplements. JTD owns stock in several food and drug companies. JTD reports board memberships with the McCormick Spice Institute, The Mushroom Council, Bay State Milling Company, the Singapore Institute of Food and Biotechnology Innovation (SIFB), the International Life Sciences Institute, and *Nutrition Today*. She also reports consulting or advisory roles with Nestlé and the Institute for the Advancement of Food and Nutrition Sciences. All other authors report no conflicts of interest.
