# Supplementary material for: Methods matter for dietary supplement exposure assessment: comparing prevalence, product types, and amounts of nutrients from dietary supplements in the Interactive Diet and Activity Tracking in the American Association of Retired Persons cohort study
Source: Am J Clin Nutr. 2025 Apr 2;121(6):1258–67. doi: 10.1016/j.ajcnut.2025.03.020 (PMC12226753; doi:10.1016/j.ajcnut.2025.03.020)
Supplement: Multimedia component 1 [file mmc1.docx]

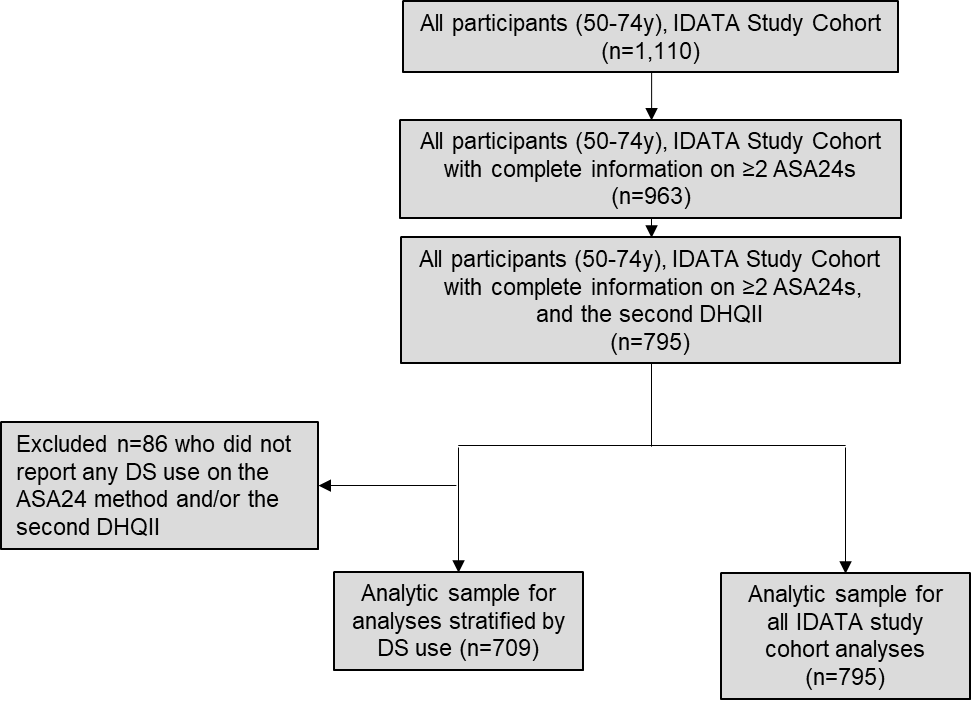


Supplemental Figure 1. Participant flowchart outlining sample identification in the current investigation using IDATA cohort study data

Supplemental Table 1. Prevalence of use and agreement of non-micronutrient-containing product types on the ASA24 and DHQII methods among the IDATA study sample (N=795)^1^

|  | Total Sample | Group-Level Prevalence on ASA24 | Group-Level Prevalence on DHQII | Individual-Level Agreement | Comparison of Group Level Prevalence |
| --- | --- | --- | --- | --- | --- |
|  | n | n (%) | n (%) | Kappa^2^ | *p*-value^3^ |
| *Product Type* |  |  |  |  |  |
| Eye Health Product | 795 | 34 (4.3) | 38 (4.8) | 0.53 | 0.480 |
| Botanical | 795 | 134 (16.9) | 115 (14.5) | 0.49 | 0.066 |
| Coenzyme Q10 | 795 | 81 (10.2) | 82 (10.3) | 0.71 | 0.879 |
| Energy Supplement | 795 | 19 (2.4) | 9 (1.1) | -0.02 | 0.059 |
| Omega 3 | 795 | 336 (42.3) | 253 (31.8) | 0.65 | <0.001 |
| Fish Oil | 795 | 335 (42.1) | 241 (30.3) | 0.64 | <0.001 |
| Flaxseed Oil | 795 | 6 (0.8) | 50 (6.3) | 0.17 | <0.001 |
| Joint Product | 795 | 163 (20.5) | 99 (12.5) | 0.58 | <0.001 |
| Probiotics | 795 | 38 (4.8) | 64 (8.1) | 0.60 | <0.001 |
| Sports Supplement | 795 | 36 (4.5) | 6 (0.8) | -0.01 | <0.001 |

Abbreviations: DS, Dietary Supplement; DHQII, Diet History Questionnaire-II; ASA24, Automated Self-Administered 24-hour dietary recall.

^1^ Unless otherwise indicated, values are percentages (%). The analytic sample includes individuals aged 50-74y who had complete information for the second DHQII and ASA24s.

^2^ Kappa (κ) reflects the agreement of DS product types on the ASA24 and the DHQII at the individual level. The κ was interpreted as follows: values ≤ 0 (no agreement); 0.01–0.20 (none to slight agreement), 0.21–0.40 (fair agreement), 0.41– 0.60 (moderate agreement), 0.61–0.80 (substantial agreement), and 0.81–1.00 (almost perfect agreement).

^3^ *p*-value indicates a statistically significant difference in estimated group-level prevalence (i.e., via McNemar’s test) between the DHQII and ASA24 methods. A Bonferroni-corrected *p*-value<0.00625 was considered statistically significant.

Supplemental Table 2. Estimated percent (%) of IDATA dietary supplement users who are taking selected nutrients from all product types or a multivitamin-mineral, by sex and DS assessment method (n=709)^1^

|  | From All Product Types | | | |  |
| --- | --- | --- | --- | --- | --- |
|  | Calcium (mg/d) | | Vitamin D (µg/d) | |  |
|  | *n* | % | *n* | % |  |
| DHQ only |  |  |  |  |  |
| *Males (n=347)* | 254 | 73.2 | 286 | 82.4 |  |
| *Females (n=362)* | 298 | 82.3 | 327 | 90.3 |  |
| ASA24 |  |  |  |  |  |
| *Males (n=347)* | 265 | 76.4 | 279 | 80.4 |  |
| *Females (n=362)* | 297 | 82.0 | 307 | 84.8* |  |
|  | From MVM | | | |  |
| DHQ only |  |  |  |  |  |
| *Males (n=347)* | 235 | 67.7 | 235 | 67.7 |  |
| *Females (n=362)* | 226 | 62.4 | 226 | 62.4 |  |
| ASA24 |  |  |  |  |  |
| *Males (n=347)* | 238 | 68.6 | 234 | 67.4 |  |
| *Females (n=362)* | | 230 | 63.5 | 227 | 62.7 |

Abbreviations: DS, Dietary Supplement; DHQII, Diet History Questionnaire-II; ASA24, Automated Self-Administered 24-hour dietary recall; MVM, multivitamin-mineral; SE, standard error.

^1^ Unless otherwise indicated, values are percentages (%). The analytic sample includes individuals aged 50-74y who had complete information for the second DHQII and ASA24s and reported DS intake on the DHQII or an ASA24. An asterisk (*) indicates a statistically significant difference in the estimated prevalence of IDATA DS users who are taking selected nutrients from any DS or a specific DS product type, when compared with the estimated prevalence of the referent group (i.e., DHQ only), within sex. McNemar’s test by sex was used to identify statistical significance at *P*<0.05.

**Appendix 1: Supplemental Methods**

*Eligibility Criteria for IDATA Study*

AARP members who were 50-74 years and literate in the English language, had access to high-speed internet, were reasonably mobile (physically), were free of health conditions affecting metabolism, did not have prior formal nutrition training, and were not on a weight loss diet were considered eligible (1, 2). Eligible study participants visited the study center, and informed consent was obtained from all participants with confirmed eligibility; only one AARP member per household was considered eligible (n=1,130) (1, 2). An additional 20 individuals were also excluded due to lack of information, resulting in a final sample of 1,110 men and women aged 50 to 74 years who participated in the IDATA cohort study.

*DS Product Specific Questions and Nutrient Amounts on the DHQII*

Participants reported frequency of use of multivitamin, calcium, iron, and vitamins C and E products using the options: “< 1 day per month,” “1-3 days per month,” “1-3 days per week,” “4-6 days per week,” or “every day.” Participants were also queried on long-term use of these five products, and asked to report the number of years the product had been taken (options ranging from: <1 year to 10 or more years). For calcium and vitamins C and E, information on dose (i.e., nutrient amount on the product label) was collected, with options corresponding to the nutrient; calcium: “<500 mg”, “500-599 mg”, “600-699 mg”, “≥1,000 mg”, “don’t know”; vitamin C: “500 mg”, “500-999 mg”, “1,000-1,499 mg”, “1,500-1,999 mg”, “≥2,000 mg,” “don’t know”; vitamin E: “< 400 IU”, “400-799 IU”, “800-999 IU”, “≥1,000 IU”, “don’t know” (3). Frequencies of intake reported on the DHQ II were summed into daily frequencies and multiplied by the amounts of nutrients reported per dose. Nutrient intakes were estimated using the USDA Food and Nutrient Database for Dietary Studies (versions 1.0-3.0) and the MyPyramid Equivalents Database (version 2.0) for intakes from foods and beverages, and the NHANES DSD for intakes from DS.

*Calculation of Mean Nutrient Intakes and Mean Intakes Per Consumption Day from DS*

On the ASA24, mean calcium and vitamin D intake from DS was calculated using the number of days of reported DS use out of the total number of completed recalls (ranges from 2 to 6 recalls), multiplied by the nutrient amount the participant reported taking per day. All calcium and vitamin D dosages were assigned by the product label on the ASA24. On the DHQII, mean intakes from DS were computed using the number of days of DS use over the previous 12 months, multiplied by the nutrient amount the participant reported taking per day. Default values for calcium and vitamin D were assigned according to the product type on the DHQII; for calcium, an MVM was assumed to include 162 mg of calcium, whereas non-MVM sources of calcium on the DHQII were assigned based on the dosage categories reported (as described above). Because vitamin D intake amounts from DS were not queried on during the DHQII, an MVM, multivitamin, or single vitamin D product was assumed to contain 10 micrograms of vitamin D; whereas, for calcium and vitamin D products, the default value was 5 micrograms of vitamin D.

**REFERENCES**

1. Park Y, Dodd KW, Kipnis V, et al. Comparison of self-reported dietary intakes from the Automated Self-Administered 24-h recall, 4-d food records, and food-frequency questionnaires against recovery biomarkers. *Am J Clin Nutr* 2018;107(1):80-93.

2. Subar AF, Potischman N, Dodd KW, et al. Performance and Feasibility of Recalls Completed Using the Automated Self-Administered 24-Hour Dietary Assessment Tool in Relation to Other Self-Report Tools and Biomarkers in the Interactive Diet and Activity Tracking in AARP (IDATA) Study. *J Acad Nutr Diet* 2020;120(11):1805-20.

3. National Cancer Institute. Diet History Questionnaire II (DHQ II) for U.S. & Canada. (<https://epi.grants.cancer.gov/dhq2/>). (Accessed February 9, 2022).
